# Heme Oxygenase 1 Impairs Glucocorticoid Receptor Activity in Prostate Cancer

**DOI:** 10.3390/ijms20051006

**Published:** 2019-02-26

**Authors:** Daiana B. Leonardi, Nicolás Anselmino, Javier N. Brandani, Felipe M. Jaworski, Alejandra V. Páez, Gisela Mazaira, Roberto P. Meiss, Myriam Nuñez, Sergio I. Nemirovsky, Jimena Giudice, Mario Galigniana, Adalí Pecci, Geraldine Gueron, Elba Vazquez, Javier Cotignola

**Affiliations:** 1 Laboratorio de Inflamación y Cáncer, Departamento de Química Biológica, Facultad de Ciencias Exactas y Naturales, Universidad de Buenos Aires, Buenos Aires C1428EGA, Argentina; daialeonardi@yahoo.com.ar (D.B.L.); nicoanselmino@hotmail.com (N.A.); jnbrandani@gmail.com (J.N.B.); felipejaw@gmail.com (F.M.J.); alejandravpaez@gmail.com (A.V.P.); ggueron@gmail.com (G.G.); 2 Instituto de Química Biológica de la Facultad de Ciencias Exactas y Naturales (IQUIBICEN), CONICET Universidad de Buenos Aires, Buenos Aires C1428EGA, Argentina; gisela.mazaira@gmail.com (G.M.); sergio.nemirovsky@gmail.com (S.I.N.); 3 Laboratorio de Biología Celular y Molecular, Departamento de Química Biológica, Facultad de Ciencias Exactas y Naturales, Universidad de Buenos Aires, Buenos Aires C1428EGA, Argentina; mgali@qb.fcen.uba.ar; 4 Departamento de Patología, Instituto de Estudios Oncológicos, Academia Nacional de Medicina, Buenos Aires C1425AUM, Argentina; rpmeiss@gmail.com; 5 Departamento de Físico-Matemática, Facultad de Farmacia y Bioquímica, Universidad de Buenos Aires, Cátedra de Matemática, Buenos Aires C1113AAD, Argentina; myr1710@yahoo.com; 6 Departamento de Química Biológica, Facultad de Ciencias Exactas y Naturales, Universidad de Buenos Aires, Buenos Aires C1428EGA, Argentina; pecciadali@gmail.com; 7 Department of Cell Biology and Physiology, University of North Carolina, Chapel Hill, NC 27599-7545, USA; jimena.giudice@gmail.com; 8 McAllister Heart Institute, University of North Carolina, Chapel Hill, NC 27599-7126, USA; 9 Laboratorio de Receptores Nucleares, Instituto de Biología y Medicina Experimental (IBYME), CONICET, Buenos Aires C1428ADN, Argentina; 10 Biología Molecular y Neurociencias (IFIBYNE), Instituto de Fisiología, CONICET—Universidad de Buenos Aires, Buenos Aires C1428EGA, Argentina

**Keywords:** glucocorticoid receptor, GR, heme oxygenase 1, HO-1, prostate cancer

## Abstract

Glucocorticoids are used during prostate cancer (PCa) treatment. However, they may also have the potential to drive castration resistant prostate cancer (CRPC) growth via the glucocorticoid receptor (GR). Given the association between inflammation and PCa, and the anti-inflammatory role of heme oxygenase 1 (HO-1), we aimed at identifying the molecular processes governed by the interaction between HO-1 and GR. PCa-derived cell lines were treated with Hemin, Dexamethasone (Dex), or both. We studied GR gene expression by RTqPCR, protein expression by Western Blot, transcriptional activity using reporter assays, and nuclear translocation by confocal microscopy. We also evaluated the expression of HO-1, FKBP51, and FKBP52 by Western Blot. Hemin pre-treatment reduced Dex-induced GR activity in PC3 cells. Protein levels of FKBP51, a cytoplasmic GR-binding immunophilin, were significantly increased in Hemin+Dex treated cells, possibly accounting for lower GR activity. We also evaluated these treatments in vivo using PC3 tumors growing as xenografts. We found non-significant differences in tumor growth among treatments. Immunohistochemistry analyses revealed strong nuclear GR staining in almost all groups. We did not observe HO-1 staining in tumor cells, but high HO-1 reactivity was detected in tumor infiltrating macrophages. Our results suggest an association and crossed modulation between HO-1 and GR pathways.

## 1. Introduction

Glucocorticoid receptor (GR) activation, mediated by the binding of glucocorticoids, regulates the expression of inflammatory factors. Many chaperones are involved in the trafficking and turnover of this receptor. Cytoplasmic GR forms heterocomplexes with the Hsp90-binding immunophilin FKBP51 [1]. Upon steroid binding, FKBP51 is released from GR and replaced by FKBP52 which, in turn, recruits dynein/dynactin [1]. Therefore, this immunophilin exchange assemblies the molecular machinery for the efficient and fast nuclear transport of GR.

In prostate cancer (PCa), glucocorticoids are usually used to counteract the pain associated with bone metastasis and the toxic effects triggered by therapy. On the other hand, GR is upregulated in castration-resistant tumors and its over-expression is involved in the development of abiraterone and enzalutamide resistance [2], bypassing androgen receptor (AR) blockage and regulating a subset of distinguishable AR target genes [3]. The GR upregulation can be explained by the lack of AR binding to a negative androgen response element in the GR promoter [4].

Heme oxygenase 1 (HO-1), the rate limiting enzyme of heme degradation, is of vital importance in the inflammatory response [5]. Although in many tumors its expression has been associated with progression and increased aggressiveness, our group showed that high HO-1 expression inhibits PCa cell proliferation, migration, and invasion in vitro [6], and prevents tumor growth and angiogenesis in vivo [6,7]. Similar results were reported for other tumors [5]. We also showed that HO-1 can negatively modulate AR transcriptional activity by interfering with STAT3 signaling [8]. Additionally, HO-1 exhibits nuclear localization in different tumors [9,10,11,12]; however, the role of nuclear HO-1 is still unknown.

The current literature on HO-1 and GR association is controversial and non-conclusive. Lutton et al. showed that HO-1 was under-expressed when hepatocarcinoma cells were treated with Dexamethasone [13]. Additionally, Lavrovsky et al. revealed the existence of a negative glucocorticoid response element in the HO-1 promoter [14] and that glucocorticoid-treated endothelial cells had lower HO-1 expression [15]. On the other hand, an upregulation of HO-1 by Dexamethasone was shown in monocytes [16] and larynx carcinoma cells [17].

The aim of this study was to analyse the interaction between GR and HO-1 and to identify the molecular processes governed by the association between these two proteins in PCa.

## 2. Results

### 2.1. NR3C1 Expression and Signaling is Modulated by Hemin Treatment

**Figure 1:**
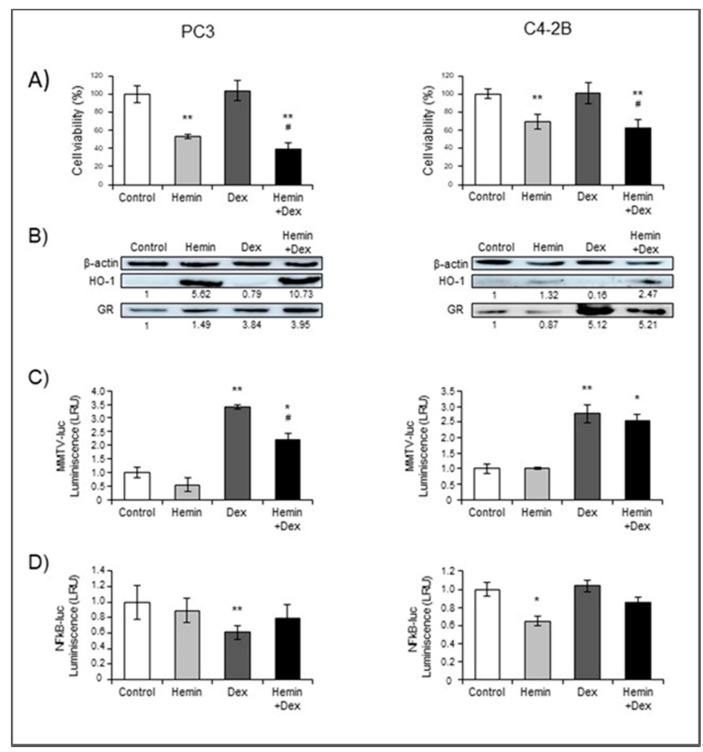
Hemin treatment modulates Dexamethasone-induced GR expression and signaling. PC3 and C4-2B cells were treated with Hemin (80 μM for 24 h), Dexamethasone (Dex; 10 nM for 6 h post Hemin/PBS 24-h treatment), the combination of both drugs, or PBS as control. (**A**) MTS viability assay was performed and results are presented as percentage of viable cells compared to control (100%). (**B**) Western blot analysis showing HO-1, GR, and β-actin as loading control. Protein quantification was performed by densitometry analysis using ImageJ software. The numbers under the bands indicate the quantitation normalized to β-actin and control lane. One representative experiment is shown. Panels C and D depict reporter assays. Cell lines were transiently transfected with the MMTV-luc (**C**) or NFkB-luc (**D**) reporter plasmids, and after treatments, cells were lysed and luciferase activity assay was performed. Data were normalized to total protein values. Results are shown as mean ± SEM from at least three independent experiments; * *p* < 0.05 and ** *p* < 0.01 versus control cells, # *p* < 0.05 versus Dex treated cells.

We first analyzed whether glucocorticoids or hemin treatments affected PC3 (GR+/AR−) and C4-2B (GR+/AR+) viability. Hemin, a potent inducer of HO-1, significantly decreased the viability of both cell lines, either in the presence or absence of Dexamethasone (Figure 1A). Hemin-induced HO-1 over-expression was confirmed by Western Blot and RTqPCR (Figure 1B and Supplemental Figure S1, respectively). *NR3C1* (GR gene) mRNA expression was significantly decreased under Hemin and Hemin+Dexamethasone treatments in both cell lines, while it did not change under Dexamethasone treatment (Supplemental Figure S1). However, we observed that GR protein levels were significantly elevated by more than 3-fold in cells exposed to Dexamethasone (Figure 1B). The higher protein levels detected, even when *NR3C1* mRNA levels were similar or lower to control, might be due to lower GR proteasome degradation, as was previously demonstrated [18].

We further analyzed the expression levels of *IKBA* and *BCLXL*, two GR downstream targets, to infer GR activity. Hemin significantly downregulated the transcription of both genes in PC3 independently of Dexamethasone treatment (Supplemental Figure S1), while in C4-2B this repressive effect was observed only on the Dexamethasone-induced condition (Supplemental Figure S1).

In order to further investigate the transcriptional activity of GR, we used a reporter plasmid (MMTV-luc) with GR response elements. Dexamethasone-induced luciferase activity was confirmed in both cell lines (Figure 1C), but hormone-activation was partially abrogated in PC3 cells pre-treated with Hemin (Figure 1C, left panel), albeit the protein levels were similar to those in Hemin-alone treated cells. Additionally, we studied the GR-mediated transrepression activity on NFkB pathway, using a luciferase reporter plasmid (NFkB-luc) that responds to this pro-inflammatory factor [7]. Dexamethasone inhibited luciferase activity in PC3 cells, thus reflecting the higher GR-transrepressive effect provoked by the glucocorticoid (Figure 1D), which was reversed when cells were pre-treated with Hemin. This effect was not observed in C4-2B (Figure 1D). However, in this cell line, Hemin reduced NFkB-luc activity under the experimental conditions assayed (Figure 1D).

**Figure 2:**
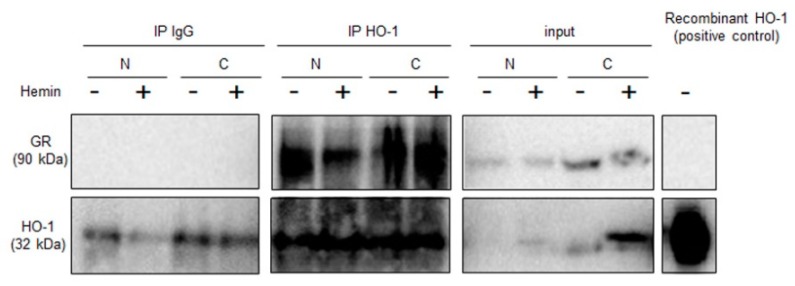
HO-1 associates to GR in PC3 cells. PC3 cells were treated with Hemin (80 μM, 24 h) or PBS as control. Nuclear and cytoplasmic compartments were isolated. Cell extracts were immunoprecipitated using an anti-HO-1 polyclonal antibody or IgG as negative control. Complexes were analysed by SDS-polyacrylamide gel electrophoresis and immunoblot assay with anti-GR and anti-HO-1 antibodies.

Given our previous data showing that HO-1 interacts with and modulates STAT3 [8], a critical transcription factor in PCa, we sought to test whether a direct protein association exists between GR and HO-1. Co-immunoprecipitation assays in PC3 cells suggested that HO-1 and GR interact (Figure 2).

**Figure 3:**
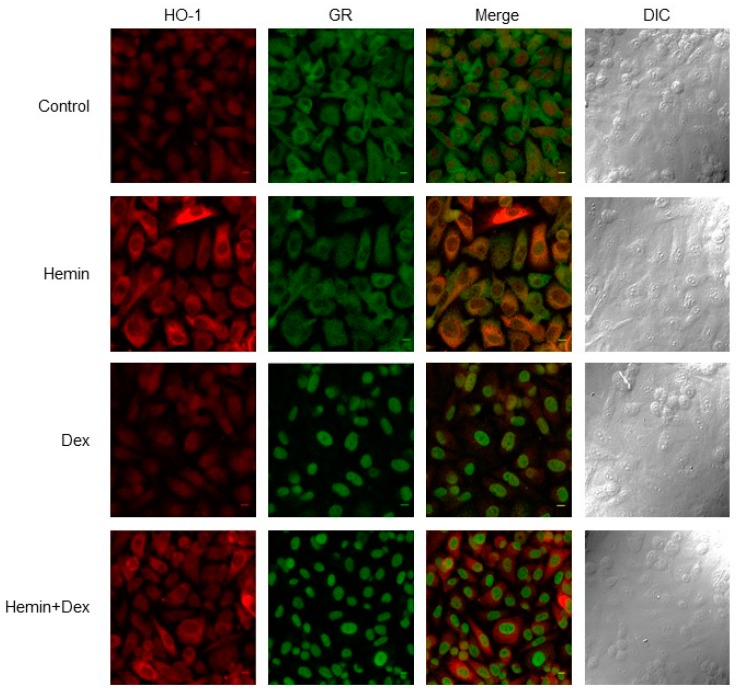
Analysis of HO-1 and GR subcellular localization. Immunofluorescence analysis of HO-1 and GR expression and localization in PC3 cells treated with Hemin (80 μM for 24 h), Dex (10 nM for 6 h post Hemin/PBS 24-h treatment), and the combination of both drugs or PBS as control. Cells were fixed, stained with anti-HO1, and anti-GR primary antibodies and secondary antibodies conjugated with Alexa Fluor 488 (red, HO-1) and 555 (green, GR) antibodies. Cells were imaged by confocal microscopy using the same parameters for all the treatments. A representative image for each condition is shown. Final magnification: ×60.

We previously reported that Hemin treatment retained STAT3 in the cytoplasm [8], therefore, here we analyzed GR subcellular localization under HO-1 induced expression. Analysis of confocal immunofluorescence microscopy confirmed HO-1 over-expression under Hemin treatment and a higher nuclear localization of this protein (Figure 3). GR expression and its nuclear localization increased under Dexamethasone treatment, and this effect was not impaired by Hemin pre-treatment (Figure 3).

Altogether, these results suggest that HO-1 modulates GR signaling in PCa cells without interfering with GR nuclear translocation.

### 2.2. Identification of Glucocorticoid Response Elements in HO-1 Proximal Promoter

Confocal Immunofluorescent microscopy revealed altered HO-1 expression in Hemin+Dexamethasone treated cells compared to Hemin alone treatment (Figure 3). We performed an *in-silico* analysis of the HO-1 promoter region (estimated at Chr22:35379360–35380560) to identify glucocorticoid response elements (GRE). As shown in Supplemental Table S1, HO-1 proximal promoter does not contain consensus GRE sequences. However, we cannot rule out the presence of other GRE in distant regions, such as enhancers.

### 2.3. Hemin Treatment Increases FKBP51 Expression in the Presence of Dexamethasone

**Figure 4:**
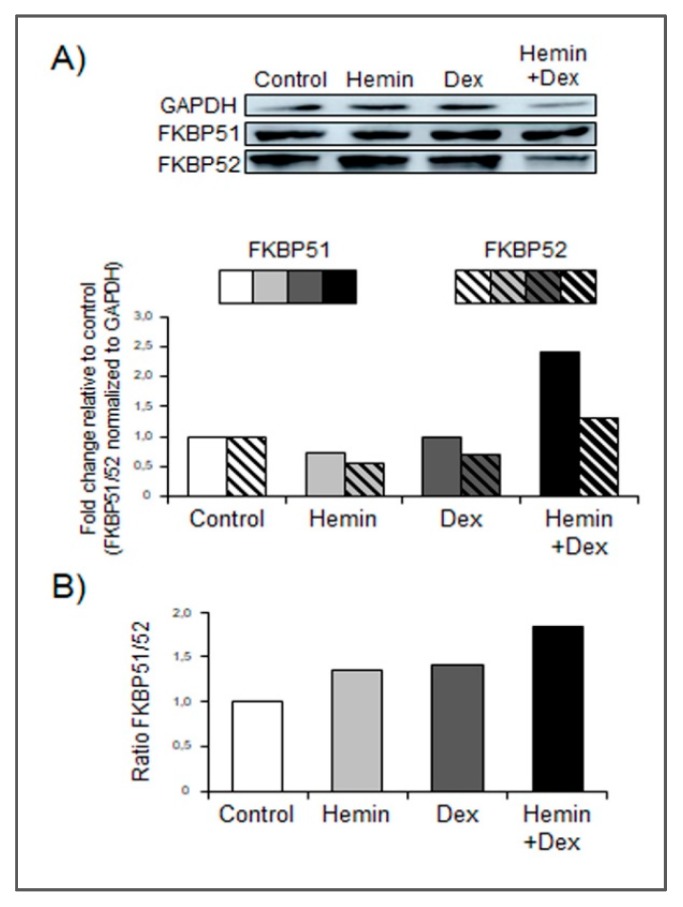
Hemin increases FKBP51 expression under Dexamethasone stimulation. (**A**) Western blot analysis showing FKBP51 and FKBP52 expression in PC3 cells treated with Hemin (80 μM for 24 h), Dex (10 nM for 6 h post Hemin/PBS 24-h treatment), the combination of both drugs, or PBS as control. Total protein was extracted and protein expression was analyzed by western blotting using specific antibodies. GAPDH levels are shown as control for equal loading. Protein quantification was performed by densitometry analysis using ImageJ software and bands were normalized to GAPDH and control. (**B**) FKBP51/FKBP52 ratio was calculated for each condition. One representative from at least three independent experiments is shown.

Strong evidence suggest that FKBP51 and FKBP52 have a role in the modulation of GR activity and glucocorticoid-dependent translocation of GR from the cytosol to the nucleus [1]. Western blot analysis revealed a significant increase of FKBP51 in cells under HO-1 induction and GR stimulation with respect to cells that received only single treatments or vehicle (Figure 4A). Furthermore, Hemin+Dexamethasone treatment triggered a higher FKBP51/52 expression ratio (Figure 4B).

### 2.4. Study of Hemin and/or Dexamethasone Treatment in PC3 Xenografts

**Figure 5:**
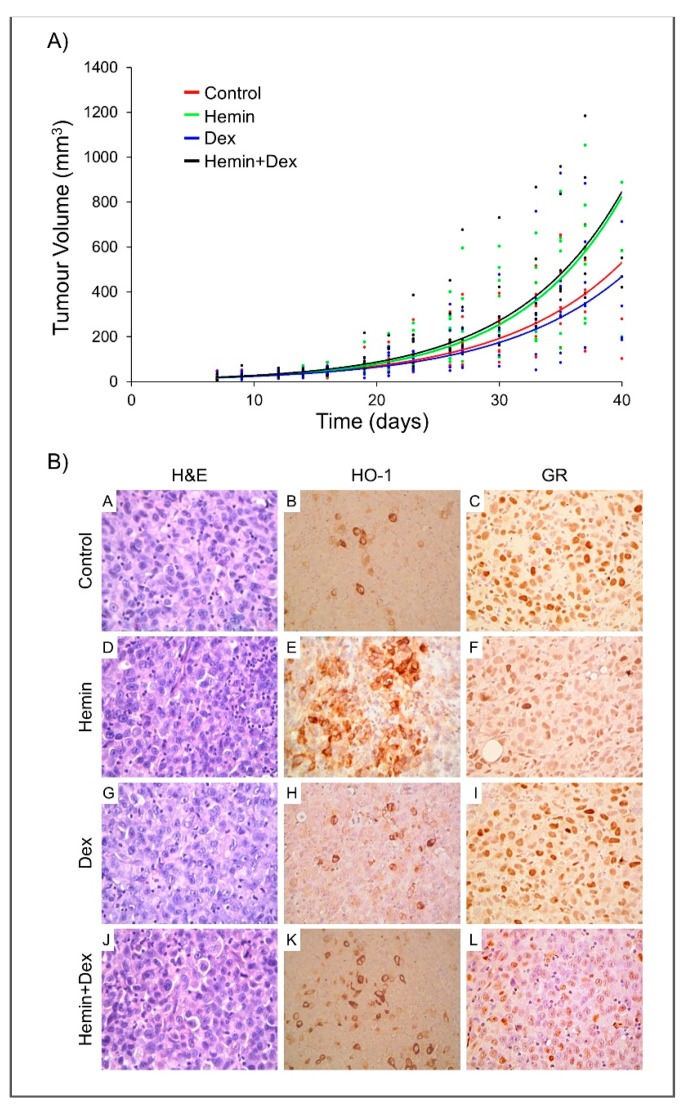
In vivo effect of Hemin and/or Dexamethasone on PC3 xenografts growth. Six- to eight-week-old male athymic nude (nu/nu) mice were randomized into four groups (*n* = 7 per group). PC3 cells (3.6 × 10^6^) were injected *s.c.* in the right flank. When tumors reached a volume of around 150 mm^3^, animals were *i.p.* injected every 48 h with 6 doses of Hemin (25 mg/kg), Dexamethasone (0.2 mg/kg), Hemin+Dexamethasone (same doses of individual treatments), or PBS (control). (**A**) Exponential regression of tumor volume was calculated for each treatment according to the volume measured, as described in Materials and Methods along the experimental procedure. (**B**) Histological (H&E, left panel) and immunohistochemical analysis of paraffin-embedded tumor sections obtained from treated or control mice at the experimental end point. HO-1 and GR immunohistochemical analysis were performed using specific antibodies: HO-1 negative immunostaining in tumor cells and positive HO-1 reactivity in macrophages (central panel) and GR-positive nuclear immunostain (right panel). Final magnification: H&E × 250, HO-1 × 250, GR × 100. One representative image of each condition is shown.

Given that Dexamethasone is frequently used as a palliative treatment in patients with PCa, and the relevance of inflammation in this disease, we used a human PCa xenograft model to investigate the effect of Hemin, Dexamethasone, or both on tumor growth. No significant alterations were observed in the body weight of the animals in the different groups (Supplemental Figure S2). For each treatment (*n* = 7), the exponential regression of tumor volume curve was plotted and duplication time for each condition was calculated (Figure 5A). Non-significant differences were observed among treatments in the tumor growth nor in *HMOX1*, *BCLXL*, *IKBA,* and *MKI67* gene expression (Supplemental Figure S2). As previously reported in vitro [19], a significant down-regulation of GR mRNA levels was detected in animals of the Dexamethasone group (Supplemental Figure S2).

Histological analysis at the time of euthanasia revealed the presence of poorly differentiated carcinomas, with irregular nuclei, prominent nucleoli, and aberrant mitotic images (Figure 5B). Immunohistochemical staining showed negative HO-1 expression in the tumor cells in all the groups. However, positive HO-1 expression was detected in macrophages in all the animals with higher immunoreactivity in Hemin, Dexamethasone, and Hemin+Dexamethasone groups (Figure 5B). Nuclear GR staining was detected in all cases; nevertheless, different intensities were observed—strong in control and Dexamethasone treated animals, weak to moderate in the Hemin group and weak in the co-treated group (Figure 5B).

### 2.5. High NR3C1 and HMOX1 Expression Reduces PCa-patient Disease-Free Survival

**Figure 6:**
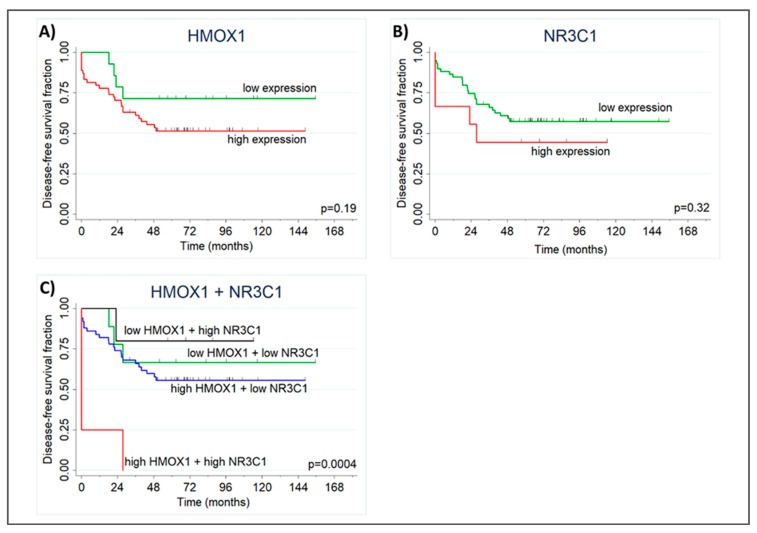
*NR3C1* and *HMOX1* high expression reduces PCa-patient disease-free survival. Kaplan Meier plot showing groups with low and high *HMOX1* (**A**) and *NR3C1* (**B**) expression according to ROC curve threshold. (**C**) Kaplan Meier plot showing groups with low and high *HMOX1* and *NR3C1* combined expression according to ROC curve threshold. Patients with high expression of both genes have shorter disease-free survival compared to other groups (log-rank *p* = 0.0004). Vertical marks show censored patients.

To evaluate whether *NR3C1* and *HMOX1* tumoral expression have clinical implications, we analyzed disease-free survival and relapse-free survival using data from public repositories. RNAseq data showed non-significant differences in survival when both genes were examined individually (Figure 6A,B). However, when the combined expression of *NR3C1* and *HMOX1* was considered, we found that patients with high expression of both genes have significantly worse disease-free survival (HR = 9.3, 95%CI = 2.0–42.9, *p* = 0.004; Figure 6C).

## 3. Discussion

In this study, we report for the first time that Hemin negatively modulates GR activity induced by Dexamethasone in PCa cells (PC3, GR+/AR−) (Figure 1). The reduced GR activity observed under Hemin+Dexamethasone treatment could be explained by the increased expression of FKBP51 (Figure 4). These findings suggest a crosstalk between HO-1 and GR pathways in PC3 cells. Furthermore, co-immunoprecipitation analysis suggested a physical interaction between these two proteins. Overall, we demonstrated that HO-1 alters GR signaling in PCa cells without impairing GR nuclear translocation.

We have previously shown the direct interaction between HO-1 and STAT3, the cytoplasmic retention of this transcription factor, and the consequent down-regulation of AR transcriptional activity [8]. HO-1 was also shown to interact with other proteins [20,21]. In this study, we demonstrated that Hemin treatment is able to impair GR activity in vitro, even though GR is able to translocate to the nucleus in the presence of Dexamethasone. This observation agrees with results already reported showing a FKBP51-dose-dependent inhibition of GR activity [22] partially mediated by the reduction of hormone binding [23]. Furthermore, it was demonstrated that in WCL2 cells that constitutively over-expresses GR, this receptor was localized in the nucleus, even without steroid stimulation [24]. Since GR activity and subcellular localization were performed at 6 h post Dexamethasone treatment, we cannot rule out that HO-1 interferes with GR nuclear translocation kinetics at shorter times (<2 h). In agreement with this, it was previously reported that geldanamycin (Hsp90 inhibitor) reduced the mineralocorticoid receptor nuclear translocation [24]. At longer times, hormone receptors could translocate to the nucleus in a cytoskeleton-independent manner.

HO-1 is one of the NRF2 downstream targets [25] and NRF2 activity is modulated by nuclear receptors, including GR [26]. It was reported that GR signaling represses NRF2-dependent transcriptional activation [27]. Accordingly, we observed that HO-1 mRNA levels were lower under Dexamethasone treatment (Figure 1B). However, Dexamethasone-mediated repression was not detected when cells were pretreated with hemin, probably due to the high levels of HO-1 reached before the glucocorticoid treatment.

Although GR and HO-1 expression profile were similar in C4-2B (GR+/AR+) and PC3 (GR+/AR−) in all treatments (Supplemental Figure S1), GR activity induced by Dexamethasone was not significantly altered in Hemin-treated C4-2B cells (Figure 1). The lack of effect could be explained by the presence of a mutant AR, as it is well known that MMTV-luc reporter plasmid can also be activated by AR [28]. Moreover, it was recently documented that PCa cells with high AR expression have lower GR dependency [29].

Dexamethasone is frequently used as a palliative treatment in patients with PCa [30]. Considering that Hemin modulates GR activity in vitro, we assessed the effect of this protoporphyrin and Dexamethasone co-treatment using PC3-derived tumors growing as xenografts. In accordance with a previous report [31], no significant differences in tumor growth was observed when animals received Dexamethasone. On the other hand, DU145 xenografts treated with peritumoral injections of Dexamethasone showed a significantly lower growth rate compared to the control group [32]. Furthermore, Hemin treatment had no effect on tumor growth when administered, either alone or in combination with the glucocorticoid. It is worth mentioning that we recently reported that Hemin conditioning prior to tumor challenge resulted in a significant increase in tumor latency compromising the tumor vascularization and the immune response [33]. However, these results were obtained using a pre-clinical model of PCa in immunocompetent mice, another PCa cell line was used, and drugs were administered with different protocols [32,33].

Immunohistochemistry analysis of PC3 xenografts revealed negative HO-1 staining in tumor cells demonstrating that Hemin *i.p.* treatment did not induce HO-1 expression in tumor epithelial cells. However, we observed strong positive HO-1 immunoreactivity was detected in tumor infiltrating macrophages (Figure 5). In agreement with these results, analyses of human prostate carcinomas and benign prostatic hyperplasia samples showed HO-1 positive staining in stromal and infiltrating immune cells [11,34]. HO-1 expression was also reported in extra tumoral macrophages and associated with tumor aggressiveness [34], metastatic behavior of PC3 xenografts [35], and tumor development and progression [34]. In addition, HO-1 positivity was almost exclusively seen in macrophages at the tumor invasive front in high-grade tumors from human samples [34].

Strong nuclear heterogeneous GR immunostaining was observed in Dexamethasone-treated animals and its intensity decreased in the group receiving both agents (Figure 5). Recently, other authors reported that GR is down-regulated in PCa tissue from patients sensitive to enzalutamide and abiraterone treatment compared to normal prostate [2,3]. They also described that GR expression was higher in enzalutamide/abiraterone-resistant CRPC samples [2,3].

Finally, we analyzed RNAseq data from a public repository and found that patients with high expression of *NR3C1* and *HMOX1* have shorter disease-free survival than patients with low expression of both genes. Similarly, Puhr et al. showed that patients expressing GR high levels have shorter biochemical relapse free survival [29]. However, we cannot rule out that this result was confounded by inflammation.

In summary, we demonstrated the importance of GR signaling in PCa and provided evidence about the association between GR and HO-1. Further studies will elucidate the therapeutic potential of targeting GR/HO-1 pathways in PCa therapy.

## 4. Materials and Methods

### 4.1. In Vitro Experiments

#### 4.1.1. Cell Culture and Treatments

C4-2B and PC3 cells were cultured in RPMI 1640 (Invitrogen, Buenos Aires, Argentina) supplemented with 10% FBS (fetal bovine serum) and antibiotics (penicillin 100 U/mL, streptomycin 100 μg/mL, and amphotericin 0.5 μg/mL). Cultures were maintained at 37 °C in a humidified incubator with a 5% CO_2_.

Cells were incubated 24 h in RPMI media containing 10% charcoaled FBS before they were exposed to either Hemin (80 μM, 24 h), Dexamethasone (10 nM, 6 h post Hemin/PBS treatment), the combination of both drugs, or PBS as control. Dexamethasone and Hemin were purchased from Sigma-Aldrich (St. Louis, MO, USA).

Cell viability was measured using CellTiter 96^®^ AQueous One Solution Cell Proliferation Assay (Promega, Madison, WI, USA).

#### 4.1.2. Transfections and Luciferase Reporter Assay

C4-2B and PC3 cells were seeded on 12-well plates (90% confluence) and transiently transfected with luciferase plasmid (MMTV-luc or NFkB-luc; 2 μg) using PEI (Polyethyilenimine, Polysciences Inc., Warrington, PA, USA; 4 μg). Luciferase activity was determined by the Luciferase Assay System (Promega, Madison, WI, USA) in a Glomax luminometer (Promega, Madison, WI, USA). Transfections were performed in triplicate and each experiment was repeated at least three times. Data were normalized to total protein determined by Bradford assay.

#### 4.1.3. RNA Isolation and Reverse Transcription–Quantitative PCR (RTqPCR)

Total RNA was isolated with Quick-Zol (Kalium technologies, Argentina) according to the manufacturer’s protocol. The cDNAs were synthesized with RevertAid Premium First Strand cDNA Synthesis Kit (Fermentas, Waltham, MA, USA) and used for real-time PCR amplification with Taq DNA Polymerase (Invitrogen, Waltham, MA, USA) in a Stratagene MX3000P (Agilent Technologies, Santa Clara, CA, USA). Primers sequences (5′->3′) used were: *NR3C1* (GR): TAT CTC GGC TGC GGC GGG AA and AGC GAC AGC CAG TGA GGG TGA; *HMOX1* (HO-1): ACT GCG TTC CTG CTC AAC AT and GGG GCA GAA TCT TGC ACT TT; *IkB:* ACC ATG GAA GTG ATC CGC CAG G and AGC TCC CAG AAG TGC CTC AGC AA; *PPIA:* CCC ATT TGC TCG CAG TAT CCT AGA and GGC ATG GGA GGG AAC AAG GAA AAC; *BCLXL:* GGT ATT GGT GAG TCG GAT CG and TTC CAC AAA AGT ATC CCA GC; *MKI67:* GCC AGC ACG TCG TGT CTC AAG AT and ACA CTG TCT TTT GAG TCA TCT GCG G.

Data were analyzed by Mx3000P software and normalized to the reference gene *PPIA* and control group. Data were analyzed using de 2^−ΔΔ*C*t^ method [36].

#### 4.1.4. Immunoblot Analysis and Antibodies

Total cell lysates and immunoblot analysis were carried out as previously described [6]. Briefly, cells were lysed with RIPA buffer (Tris HCl 50 mM pH 7.4; NaCl 150 mM; EDTA 20 mM pH 8; sodium deoxycholate 1%; SDS 0.1%; Triton X-100 1%, 1 mM Na_3_VO_4_, 20 mM NaF and 1 mM Na_4_P_2_O_7_, pH 7.9) and homogenized. After 20 min of incubation at 4 °C, the lysates were centrifuged at 12,000× *g* for 20 min at 4 °C and the supernatant kept at −80 °C. Lysates containing equal amounts of proteins (50 μg) were resolved on 7.5–12.5% SDS–PAGE depending on the molecular weight of the proteins under study. PageRuler Plus Prestained Protein Ladder (Fermentas, Waltham, MA, USA) was used for the estimation of molecular weight. The proteins were blotted to a Hybond-ECL nitrocellulose membrane (GE Healthcare, Little Chalfont, UK). Membranes were blocked with 5% dry non-fat milk in TBS containing 0.1% Tween 20 (TBST) for 1 h at room temperature, and incubated with primary antibodies diluted in TBST for 1 h at room temperature. Membranes were then incubated with horseradish peroxidase-labelled secondary antibody for 1 h at room temperature.

The following antibodies were used: monoclonal anti–HO-1 (catalogue 13248. Abcam, UK), monoclonal anti-GR (catalogue 12041. Cell Signaling, Danvers, MA, USA), monoclonal anti-FKBP51 (catalogue PA1-020, Affinity BioReagents, Golden, CO, USA), polyclonal anti-FKBP52 (catalogue UP30. Pharmacia and Upjohn, Inc., Pfizer, New York, NY, USA), anti–β-actin antibody (catalogue 3700. Cell Signaling, Danvers, MA, USA), and anti-mouse and anti-rabbit secondary antibodies (catalogue 7076S and catalogue 7074, respectively. Cell Signaling, Danvers, MA, USA).

#### 4.1.5. Co-Immunoprecipitation

PC3 cells were treated as described above and harvested in lysis buffer (20 mM Tris HCl, pH 8; 137 mM NaCl; 10% glicerol; 1% Triton X-100; 2 mL de EDTA 0.5 M, 1x protease inhibitor mixture (Sigma-Aldrich, St. Louis, MO, USA), 100 μg/mL PMSF, 20 mM NaF, and 1 mM Na_3_VO_4_). Proteins (500 μg) in lysis buffer were incubated overnight at 4 °C with 8 μg of anti–HO-1 antibody. Protein G Agarose beads (Invitrogen, Waltham, MA, USA) were added to each tube for 3 h at 4 °C. Beads were washed with ice-cold lysis buffer. Fifty micrograms of the lysate was used as input. Immune complexes were analyzed by immunoblot with anti-GR and anti–HO-1 antibodies.

#### 4.1.6. Immunofluorescence and Microscopy

Cells were seeded in 12-well plates at a density of 1 × 10^5^ cells per well on coverslips overnight. Cells were treated as described above and were fixed in ice-cold methanol and permeabilized for 10 min with 0.5% Triton X-100/PBS, washed with PBS, and then blocked with 5% BSA/PBS. Cells were incubated overnight with primary antibodies diluted in 4% BSA and 0.1% Tween 20 in PBS. Cells were then washed with PBS and incubated with fluorescent secondary antibodies Alexa Fluor 488 goat anti-mouse and Alexa Fluor 555 goat anti-rabbit antibodies were from Molecular Probes (Invitrogen, Waltham, MA, USA). Negative controls were carried out using PBS instead of primary antibodies. Cells were washed, mounted, and imaged by confocal laser scanning microscopy, which was performed with an Olympus Fluo view FV 1000 microscope, using an Olympus 60x/1.20 NA UPLAN APO water immersion objective.

#### 4.1.7. Statistical Analysis

GraphPad Prism software was used for statistical analysis and results are shown as mean ± standard error (SEM) of at least three independent experiments, unless stated otherwise. Student’s *t*-test or Mann-Whitney U-test were used to compare two experimental groups. For multiple comparisons, one-way ANOVA tests were performed.

### 4.2. In Vivo Experiments

#### 4.2.1. Human PCa Xenograft Model

Six- to eight-week-old male athymic nude (nu/nu) mice, each weighing at least 20 g, were purchased from Cátedra de Animales de Laboratorio y Bioterio of Facultad de Ciencias Veterinarias, UNLP, La Plata, Argentina. Mice were used in accordance with the Guidelines for the Welfare of Animals in Experimental Neoplasia [37]. The protocol was approved by the Ethical Committee (CICUAL Nº46). Mice were randomly assigned to four groups of seven animals each: Hemin, Dexamethasone, Hemin+Dexamethasone, or control (PBS). PC3 cells (3.6 × 10^6^ in 200 μL of RPMI) were injected *s.c.* (subcutaneously) in the right flank of mice using a 22G needle. Tumors were measured with a caliper starting at day 8 after inoculation, when the tumors became detectable under the skin. Tumor volumes were calculated using the formula (length × width^2^)/2. When tumors reached a volume around 150 mm^3^, the animals were *i.p.* (intraperitoneal) injected every 48 h with 6 doses of the following treatments: Hemin (25 mg/kg), Dexamethasone (0.2 mg/kg), Hemin+Dexamethasone (same doses that individual treatments), or PBS (control). Animals were sacrificed 24 h after the last dose. Resected tumors were divided, one piece was immediately placed in Quick-Zol (Kalium Technologies SRL, Bernal, Buenos Aires, Argentina) for RNA isolation, and the remaining piece was fixed in PFA 10% for immunohistochemistry staining.

#### 4.2.2. Immunohistochemical Analyses

Immunohistochemical techniques were performed as previously described [6,11]. Briefly, immunohistochemistry was done using the streptavidin-biotin-peroxidase complex system LSAB + kit, horseradish peroxidase (DAKO, Santa Clara, CA, USA). Endogenous peroxide activity was quenched using hydrogen peroxide in distilled water (3%). Antigen retrieval was done by microwaving. Tissue slides were incubated overnight with GR and HO-1 primary antibodies. This was followed by sequential incubations with biotinylated antibody and peroxidase-labelled streptavidin complex. The peroxidase reaction was conducted, under microscope, using 3,3′-diaminobenzidine. Slides were counterstained with Mayer’s hematoxylin and analyzed by standard light microscopy. Negative control slides were prepared by substituting primary antiserum with PBS. For semiquantitative analysis, the degree of staining was scored as high, moderate, low, or not detectable (3+, 2+, 1+, and 0, respectively); staining localization was also recorded. We considered positive expression when more than 10% cells exhibited positive staining.

#### 4.2.3. Statistical Analysis

To analyse the difference between treatments and experiments, we used a three-factor analysis of variance with repeated measures in the time factor. The considered factors were: treatment, time, and inter-experiment variation. Tumor volume was used as dependent variable. Wilcoxon test was employed to determine if there were differences in tumor growth among treatments.

### 4.3. In Silico Analyses

#### 4.3.1. HMOX1 Promoter Analysis

Promoter sequence was estimated between positions −1000 and +200 of *HMOX1*. Gene location and sequence belonging to the GRCh38 version of the human genome were obtained from Ensembl (http://www.ensembl.org) (access on august 2015). Possible binding of GR to the promoter sequence was assessed with the command-line version of the FIMO tool from the MEME Suit [38], using DNA binding motifs from the Cistrome platform [39] (IDs: EN0252, M00205 and MC00033) and the JASPAR database [40] (IDs: MA0113.1 and MA0113.2).

#### 4.3.2. Analysis of Human Tumor Samples

The public repository from the European Bioinformatics Institute (EBI, EMBL), Wellcome Genome Campus, Hinxton, Cambridge, UK (www.ebi.ac.uk) (access on august 2016), was browsed for studies analyzing PCa tissues. One study that included transcriptome data from 100 tumors obtained by surgery was selected (E-GEOD-54460—RNAseq Analysis of Formalin-Fixed Paraffin-Embedded Prostate Cancer Tissues” [41]). The clinico-pathological variables available were: biochemical relapse, time to relapse, pre-surgical PSA levels, pathological T stage, Gleason score, and surgical margin involvement.

Kaplan-Meier curves to study disease-free survival and biochemical relapse-free survival were performed. For these analysis, we dichotomized the patients according to high or low *NR3C1* and *HMOX1* expression. We performed Receiver Operating Characteristics (ROC) curves to determine gene expression cutoffs with the best sensitivity and specificity to predict biochemical relapse. The comparison between the groups was done with the Log-rank test. We estimated the hazard ratios (HR), 95 % confidence intervals (95% IC), and *p*-values using Cox proportional hazard models. Differences between the groups were considered significant if *p*-value ≤ 0.05. Analysis were performed using Stata v14 (StataCorp, College Station, TX, USA).

## Supplementary Materials

Supplementary materials can be found at https://www.mdpi.com/1422-0067/20/5/1006/s1. Figure S1. Gene expression profile in PCa cells treated with Hemin or Dexamethasone. Figure S2. In vivo experiments. Table S1. Predicted GR binding sites in *HMOX1* promoter region.

## References

[1] Galigniana M.D., Echeverria P.C., Erlejman A.G., Piwien-Pilipuk G. (2010). Role of molecular chaperones and TPR-domain proteins in the cytoplasmic transport of steroid receptors and their passage through the nuclear pore. Nucleus.

[2] Arora V.K., Schenkein E., Murali R., Subudhi S.K., Wongvipat J., Balbas M.D., Shah N., Cai L., Efstathiou E., Logothetis C. (2013). Glucocorticoid receptor confers resistance to antiandrogens by bypassing androgen receptor blockade. Cell.

[3] Shah N., Wang P., Wongvipat J., Karthaus W.R., Abida W., Armenia J., Rockowitz S., Drier Y., Bernstein B.E., Long H.W. (2017). Regulation of the glucocorticoid receptor via a BET-dependent enhancer drives antiandrogen resistance in prostate cancer. eLife.

[4] Xie N., Cheng H., Lin D., Liu L., Yang O., Jia L., Fazli L., Gleave M.E., Wang Y., Rennie P. (2015). The expression of glucocorticoid receptor is negatively regulated by active androgen receptor signaling in prostate tumors. Int. J. Cancer.

[5] Jozkowicz A., Was H., Dulak J. (2007). Heme oxygenase-1 in tumors: Is it a false friend?. Antioxid. Redox Signal..

[6] Gueron G., De Siervi A., Ferrando M., Salierno M., De Luca P., Elguero B., Meiss R., Navone N., Vazquez E.S. (2009). Critical role of endogenous heme oxygenase 1 as a tuner of the invasive potential of prostate cancer cells. Mol. Cancer Res..

[7] Ferrando M., Gueron G., Elguero B., Giudice J., Salles A., Leskow F.C., Jares-Erijman E.A., Colombo L., Meiss R., Navone N. (2011). Heme oxygenase 1 (HO-1) challenges the angiogenic switch in prostate cancer. Angiogenesis.

[8] Elguero B., Gueron G., Giudice J., Toscani M.A., De Luca P., Zalazar F., Coluccio-Leskow F., Meiss R., Navone N., De Siervi A. (2012). Unveiling the association of STAT3 and HO-1 in prostate cancer: Role beyond heme degradation. Neoplasia.

[9] Gandini N.A., Fermento M.E., Salomon D.G., Blasco J., Patel V., Gutkind J.S., Molinolo A.A., Facchinetti M.M., Curino A.C. (2012). Nuclear localization of heme oxygenase-1 is associated with tumor progression of head and neck squamous cell carcinomas. Exp. Mol. Pathol..

[10] Hsu F.F., Yeh C.T., Sun Y.J., Chiang M.T., Lan W.M., Li F.A., Lee W.H., Chau L.Y. (2015). Signal peptide peptidase-mediated nuclear localization of heme oxygenase-1 promotes cancer cell proliferation and invasion independent of its enzymatic activity. Oncogene.

[11] Sacca P., Meiss R., Casas G., Mazza O., Calvo J.C., Navone N., Vazquez E. (2007). Nuclear translocation of haeme oxygenase-1 is associated to prostate cancer. Br. J. Cancer.

[12] Tibullo D., Barbagallo I., Giallongo C., La Cava P., Parrinello N., Vanella L., Stagno F., Palumbo G.A., Li Volti G., Di Raimondo F. (2013). Nuclear translocation of heme oxygenase-1 confers resistance to imatinib in chronic myeloid leukemia cells. Curr. Pharm. Des..

[13] Lutton J.D., da Silva J.L., Moqattash S., Brown A.C., Levere R.D., Abraham N.G. (1992). Differential induction of heme oxygenase in the hepatocarcinoma cell line (Hep3B) by environmental agents. J. Cell. Biochem..

[14] Lavrovsky Y., Drummond G.S., Abraham N.G. (1996). Downregulation of the human heme oxygenase gene by glucocorticoids and identification of 56b regulatory elements. Biochem. Biophys. Res. Commun..

[15] Deramaudt T.B., da Silva J.L., Remy P., Kappas A., Abraham N.G. (1999). Negative regulation of human heme oxygenase in microvessel endothelial cells by dexamethasone. Proc. Soc. Exp. Biol. Med..

[16] Vallelian F., Schaer C.A., Kaempfer T., Gehrig P., Duerst E., Schoedon G., Schaer D.J. (2010). Glucocorticoid treatment skews human monocyte differentiation into a hemoglobin-clearance phenotype with enhanced heme-iron recycling and antioxidant capacity. Blood.

[17] Duzgun A., Bedir A., Ozdemir T., Nar R., Kilinc V., Salis O., Alacam H., Gulten S. (2013). Effect of dexamethasone on unfolded protein response genes (MTJ1, Grp78, Grp94, CHOP, HMOX-1) in HEp2 cell line. Indian J. Biochem. Biophys..

[18] Deroo B.J., Rentsch C., Sampath S., Young J., DeFranco D.B., Archer T.K. (2002). Proteasomal inhibition enhances glucocorticoid receptor transactivation and alters its subnuclear trafficking. Mol. Cell. Biol..

[19] Silva C.M., Powell-Oliver F.E., Jewell C.M., Sar M., Allgood V.E., Cidlowski J.A. (1994). Regulation of the human glucocorticoid receptor by long-term and chronic treatment with glucocorticoid. Steroids.

[20] Gueron G., Giudice J., Valacco P., Paez A., Elguero B., Toscani M., Jaworski F., Leskow F.C., Cotignola J., Marti M. (2014). Heme-oxygenase-1 implications in cell morphology and the adhesive behavior of prostate cancer cells. Oncotarget.

[21] Paez A.V., Pallavicini C., Schuster F., Valacco M.P., Giudice J., Ortiz E.G., Anselmino N., Labanca E., Binaghi M., Salierno M. (2016). Heme oxygenase-1 in the forefront of a multi-molecular network that governs cell-cell contacts and filopodia-induced zippering in prostate cancer. Cell Death Dis..

[22] Wochnik G.M., Ruegg J., Abel G.A., Schmidt U., Holsboer F., Rein T. (2005). FK506-binding proteins 51 and 52 differentially regulate dynein interaction and nuclear translocation of the glucocorticoid receptor in mammalian cells. J. Biol. Chem..

[23] Denny W.B., Valentine D.L., Reynolds P.D., Smith D.F., Scammell J.G. (2000). Squirrel monkey immunophilin FKBP51 is a potent inhibitor of glucocorticoid receptor binding. Endocrinology.

[24] Galigniana M.D., Erlejman A.G., Monte M., Gomez-Sanchez C., Piwien-Pilipuk G. (2010). The hsp90-FKBP52 complex links the mineralocorticoid receptor to motor proteins and persists bound to the receptor in early nuclear events. Mol. Cell. Biol..

[25] Loboda A., Damulewicz M., Pyza E., Jozkowicz A., Dulak J. (2016). Role of Nrf2/HO-1 system in development, oxidative stress response and diseases: An evolutionarily conserved mechanism. Cell. Mol. Life Sci..

[26] Namani A., Li Y., Wang X.J., Tang X. (2014). Modulation of NRF2 signaling pathway by nuclear receptors: Implications for cancer. Biochim. Biophys. Acta.

[27] Kratschmar D.V., Calabrese D., Walsh J., Lister A., Birk J., Appenzeller-Herzog C., Moulin P., Goldring C.E., Odermatt A. (2012). Suppression of the Nrf2-dependent antioxidant response by glucocorticoids and 11beta-HSD1-mediated glucocorticoid activation in hepatic cells. PLoS ONE.

[28] Iguchi K., Toyama T., Ito T., Shakui T., Usui S., Oyama M., Iinuma M., Hirano K. (2012). Antiandrogenic activity of resveratrol analogs in prostate cancer LNCaP cells. J. Androl..

[29] Puhr M., Hoefer J., Eigentler A., Ploner C., Handle F., Schaefer G., Kroon J., Leo A., Heidegger I., Eder I. (2018). The Glucocorticoid Receptor Is a Key Player for Prostate Cancer Cell Survival and a Target for Improved Antiandrogen Therapy. Clin. Cancer Res..

[30] Dorff T.B., Crawford E.D. (2013). Management and challenges of corticosteroid therapy in men with metastatic castrate-resistant prostate cancer. Ann. Oncol..

[31] Tuttle R.M., Loop S., Jones R.E., Meikle A.W., Ostenson R.C., Plymate S.R. (1994). Effect of 5-alpha-reductase inhibition and dexamethasone administration on the growth characteristics and intratumor androgen levels of the human prostate cancer cell line PC-3. Prostate.

[32] Yano A., Fujii Y., Iwai A., Kageyama Y., Kihara K. (2006). Glucocorticoids suppress tumor angiogenesis and in vivo growth of prostate cancer cells. Clin. Cancer Res..

[33] Jaworski F.M., Gentilini L.D., Gueron G., Meiss R.P., Ortiz E.G., Berguer P.M., Ahmed A., Navone N., Rabinovich G.A., Compagno D. (2017). In Vivo Hemin Conditioning Targets the Vascular and Immunologic Compartments and Restrains Prostate Tumor Development. Clin. Cancer Res..

[34] Halin Bergstrom S., Nilsson M., Adamo H., Thysell E., Jernberg E., Stattin P., Widmark A., Wikstrom P., Bergh A. (2016). Extratumoral Heme Oxygenase-1 (HO-1) Expressing Macrophages Likely Promote Primary and Metastatic Prostate Tumor Growth. PLoS ONE.

[35] Nemeth Z., Li M., Csizmadia E., Dome B., Johansson M., Persson J.L., Seth P., Otterbein L., Wegiel B. (2015). Heme oxygenase-1 in macrophages controls prostate cancer progression. Oncotarget.

[36] Livak K.J., Schmittgen T.D. (2001). Analysis of relative gene expression data using real-time quantitative PCR and the 2(-Delta Delta C(T)) Method. Methods.

[37] Workman P., Balmain A., Hickman J.A., McNally N.J., Rohas A.M., Mitchison N.A., Pierrepoint C.G., Raymond R., Rowlatt C., Stephens T.C. (1988). UKCCCR guidelines for the welfare of animals in experimental neoplasia. Lab. Anim..

[38] Bailey T.L., Boden M., Buske F.A., Frith M., Grant C.E., Clementi L., Ren J., Li W.W., Noble W.S. (2009). MEME SUITE: Tools for motif discovery and searching. Nucleic Acids Res..

[39] Liu T., Ortiz J.A., Taing L., Meyer C.A., Lee B., Zhang Y., Shin H., Wong S.S., Ma J., Lei Y. (2011). Cistrome: An integrative platform for transcriptional regulation studies. Genome Biol..

[40] Sandelin A., Alkema W., Engstrom P., Wasserman W.W., Lenhard B. (2004). JASPAR: An open-access database for eukaryotic transcription factor binding profiles. Nucleic Acids Res..

[41] Long Q., Xu J., Osunkoya A.O., Sannigrahi S., Johnson B.A., Zhou W., Gillespie T., Park J.Y., Nam R.K., Sugar L. (2014). Global transcriptome analysis of formalin-fixed prostate cancer specimens identifies biomarkers of disease recurrence. Cancer Res..

