# Supplementary material for: Heme Oxygenase 1 Impairs Glucocorticoid Receptor Activity in Prostate Cancer
Source: Int J Mol Sci. 2019 Feb 26;20(5):1006. doi: 10.3390/ijms20051006 (PMC6429053; doi:10.3390/ijms20051006)
Supplement: Supplementary file 1 [file ijms-20-01006-s001.zip › Supplemental Table 1.docx]

Supplemental Table 1

| Gene | Start | End | Strand | Score | *p*-value | *q*-value | Sequence found |
| --- | --- | --- | --- | --- | --- | --- | --- |
| *HMOX1* | 885 | 900 | - | 10,89 | 3,99x10^-5^ | 0,663 | GGCACATTCAGGTCTG |
| *HMOX1* | 320 | 337 | - | 11,29 | 5,05x10^-5^ | 0,919 | AAAAAAAAAACTTCCTGG |
| *HMOX1* | 256 | 273 | + | 10,59 | 7,20x10^-5^ | 0,919 | AGGATGATCCTTTCCAAT |
| *HMOX1* | 885 | 900 | + | 10,79 | 7,20x10^-5^ | 0,685 | CAGACCTGAATGTGCC |
| *HMOX1* | 1003 | 1018 | - | 10,53 | 8,54x10^-5^ | 0,695 | GAGGAGACTAAGTCCC |
